# Hes1 Oscillations Contribute to Heterogeneous Differentiation Responses in Embryonic Stem Cells

**DOI:** 10.3390/genes2010219

**Published:** 2011-02-22

**Authors:** Taeko Kobayashi, Ryoichiro Kageyama

**Affiliations:** 1 Institute for Virus Research, Kyoto University, Shogoin-Kawahara, Sakyo-ku, Kyoto 606-8507, Japan; E-Mail: rkageyam@virus.kyoto-u.ac.jp; 2 Japan Science and Technology Agency, CREST, Honcho, Kawaguchi-shi, Saitama 332-0012, Japan

**Keywords:** ES cell, heterogeneity, differentiation, Hes1, oscillation

## Abstract

Embryonic stem (ES) cells can differentiate into multiple types of cells belonging to all three germ layers. Although ES cells are clonally established, they display heterogeneous responses upon the induction of differentiation, resulting in a mixture of various types of differentiated cells. Our recent reports have shown that Hes1 regulates the fate choice of ES cells by repressing Notch signaling, and that the oscillatory expression of Hes1 contributes to various differentiation responses in ES cells. Here we discuss the mechanism regulating the intracellular dynamics in ES cells and how to trigger the lineage choice from pluripotent ES cells.

## 1. Introduction

Embryonic stem (ES) cells are pluripotent stem cells derived from the inner cell mass (ICM) of blastocyst stage embryos, and these cells have the ability to differentiate into various cell types belonging to all three germ layers: ectoderm, mesoderm and endoderm [1]. Application of these differentiated cells is highly anticipated for regenerative medicine, but ES cells respond heterogeneously to differentiation cues, resulting in a mixture of various types of differentiated cells [1,2]. Unexpected contamination of undifferentiated cells is also troublesome and tumorigenic *in vivo* after transplantation [1]. The basic mechanism governing such heterogeneity in ES cells is not well understood. Recent studies have revealed that the expression of Hes1, Nanog, Rex1 and other factors fluctuate in ES cells, and that ES cells expressing different levels of these factors seem to display different propensities for differentiation [3–6]. We discuss here how these fluctuations contribute to the biological output of ES cells during differentiation.

## 2. Cyclic Gene Hes1

**Figure 1:**
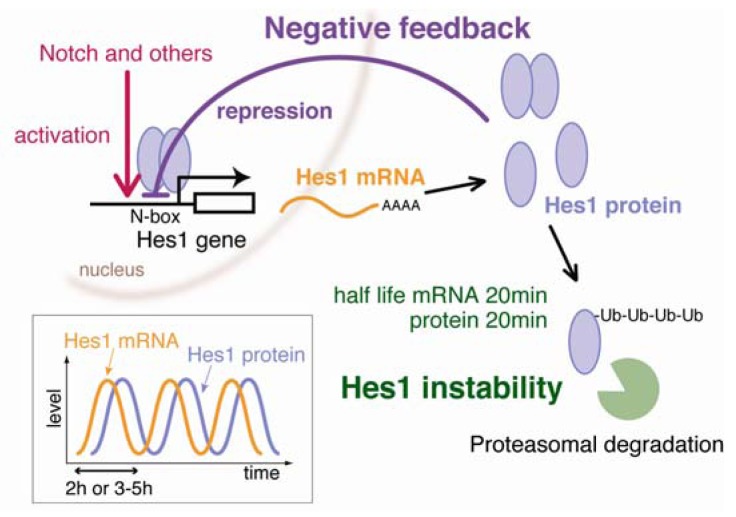
Hes1 gene expression oscillation is regulated by negative feedback and instability of gene products. Activation of the Hes1 promoter (red) induces synthesis of both Hes1 mRNA (orange) and protein (blue). Hes1 protein then binds to N box sequences of the Hes1 promoter and represses its own expression (purple). This repression leads to disappearance of Hes1 mRNA and Hes1 protein because they are extremely unstable. Hes1 protein is degraded by the proteasome (green). Disappearance of Hes1 protein relieves negative autoregulation, allowing the next round of expression. As a result, Hes1 expression oscillates in individual cells (inlet). The periodicity of Hes1 oscillation is about 2 h in fibroblast cells and about 3–5 h in ES cells.

Hes1, a member of the *Hes* gene family, encodes a basic helix-loop-helix (bHLH)-type transcriptional repressor that possesses a bHLH domain in the *N*-terminal region for DNA binding and a WRPW motif at the *C*-terminus for recruiting co-repressors [7]. Hes1 functions as a canonical effector of Notch signaling and regulates many biological events by repressing the expression of target genes that regulate differentiation [7]. Hes1 can repress its own expression by directly binding to *N*-box target sequences in its own promoter, thus forming a negative feedback loop (Figure 1). This negative feedback produces oscillating Hes1 gene expression. Hirata *et al.* demonstrated the oscillating expression of Hes1 with a period of 2 h in various cells, such as cultured fibroblasts [8]. In the developing nervous system, Hes1 oscillation is important for the maintenance and proliferation of neural stem cells under the control of Notch signaling [9,10].

## 3. Oscillatory Expression of Hes1 in Mouse ES Cells

**Figure 2:**
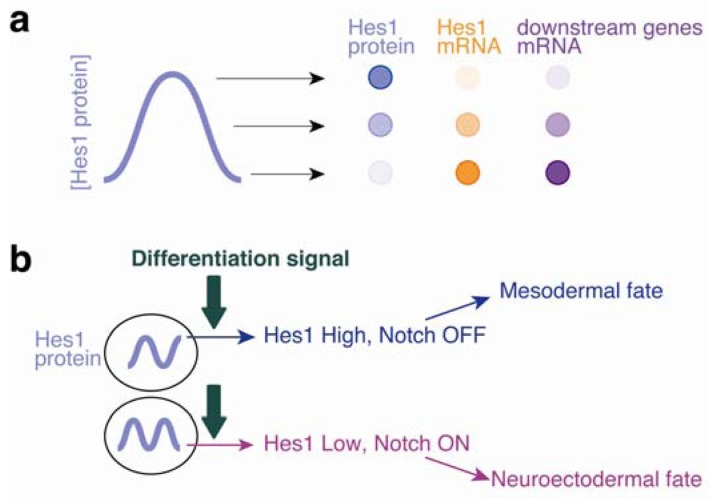
Hes1 oscillation sets heterogeneous properties in ES cells. (**a**) Hes1 protein (blue) represses mRNA synthesis of both Hes1 (orange) and Hes1 target genes (purple). Hes1 oscillation leads to dynamic changes of target-gene expression in individual ES cells; (**b**) Once differentiation signals activate ES cells, Hes1 protein-high and Hes1 protein-low level cells differentiate into early mesodermal cells (blue) and neuroectodermal cells (red), respectively, via the Notch signaling activation.

Hes1 is highly expressed in ES cells, but surprisingly the expression is not controlled by Notch signaling. Hes1 expression is under the control of bone morphogenetic protein (BMP) and leukemia inhibitory factor (LIF) [3], two factors crucial for mouse ES cell culture [11]. Hes1 expression is variable in individual ES cells, even among those in the same colony derived from a single cell. It was found that Hes1 expression oscillates in individual ES cells with a period of approximately 3–5 h, although this oscillation includes unstable fluctuations lasting shorter periods (less than 2 h) [3]. Hes1 oscillation cyclically represses the expression of both *Gadd45g* and *Dll1*, which have been identified as two major downstream targets of Hes1 by two types of gene chip analysis: expression array and chromatin immunoprecipitation (ChIP)-chip analysis. As a result, *Gadd45g* and *Dll1* display dynamic changes in their expression in individual ES cells [3]. These observations support the hypothesis that Hes1 oscillation contributes to the heterogeneous differentiation responses of ES cells by inducing oscillatory expression of genes involved in stem cell differentiation, such as the cell cycle inhibitor *Gadd45g* and the Notch signal ligand *Dll1* (Figure 2a).

Hes1 oscillations contain various modes of expression dynamics, for example, a stable oscillation, an unstable oscillation with increasing signal intensity or a stochastic noise lasting a short time [3]. The correlation between these oscillation dynamics and stem cell properties remains unknown [12]. According to a mathematical model of Hes1 oscillation, it can occur cell-autonomously; thus, if Hes1 expression is induced, the oscillation can start automatically [8] and can be maintained by transcriptional and translational delay of itself [13]. However, we do not exclude the possibility that upstream signaling pathways in ES cells, such as Jak/Stat signaling and the MAP kinase pathway under the control of LIF [14], might be oscillating and might positively regulate oscillatory Hes1 expression. Previous reports have revealed that phosphorylated active forms of Stat3 and Ras-Erk oscillate after stimulation with serum or basic fibroblast growth factor (bFGF) in cultured mouse fibroblast cells with a periodicity similar to that of Hes1 oscillation [15,16], and thus these oscillatory signaling molecules could regulate Hes1 oscillation.

## 4. Heterogeneity of ES Cell Differentiation

Several studies have reported that transcription factors associated with pluripotency are expressed in a heterogeneous manner in the ES cell population. The expression levels of the homeodomain factor Nanog and the zinc finger protein Rex1 fluctuate over several days in individual ES cells [4,5]. Nanog-positive cells also appear randomly around the inner cell mass (ICM) at the early blastocyst stage of embryos and gather in the ICM at the late blastocyst stage [17], but the phenotypic differences between these Nanog-positive and Nanog-negative cells *in vivo* remain unknown [18]. Rex1 is known to be a specific marker of the ICM in murine embryos. In the case of ES cells, Nanog-negative ES cells are fragile and susceptible to differentiation [4]; and Rex1-negative ES cells correspond to epiblast and primitive ectoderm cells rather than the naïve ES cells [5]. Another factor, Stella/Dppa3, has also been reported to be heterogeneously expressed in ES cells, and Stella expression fluctuates with a period of several days in ES cells [6]. This heterogeneity of Stella expression seems to correspond to different epigenetic statuses on histone H3 between ES cells (Stella positive) and epiblast-like cells (Stella negative) [6]. Thus, the fluctuation of gene expression seems to be a common mechanism to allow heterogeneous expression of all these genes.

Interestingly, some connections between Hes1 and these genes have been demonstrated by previous analyses [3,19]. Stella is a Hes1 target gene according to our ChIP-chip analysis, and Nanog fluctuation seems to occur in-phase with Hes1 oscillation, according to our single-cell quantitative PCR analysis [3]. A previous study identified Nanog binding sites in the Hes1 promoter region by *in silico* analysis [19]. These results indicate a correlation among fluctuating expression patterns of Stella, Nanog and Hes1. However, the reported time period required to change from a Stella- or Nanog-positive population to a Stella- or Nanog-negative population is longer (more than several days) than that of Hes1 oscillation (3–5 h) [3,4,6]. This slow fluctuation of Nanog and Stella might be regulated by other molecular mechanisms, such as a stochastic transcriptional noise [20]. We speculate that Hes1 oscillation might induce small and rapid fluctuations in slow and large fluctuations of Nanog and Stella [21], but that slow and large fluctuations of Nanog and Stella might occur independently of Hes1. The heterogeneity of ES cells is probably the sum of the fluctuation dynamics with the different periodicities of these genes, but how each gene affects the other in ES cells is highly complicated and remains unknown.

## 5. Heterogeneous Differentiation Responses by Hes1 Oscillation

We examined whether Hes1 oscillations contribute to differentiation competency by using Venus-Hes1 knock-in ES cells [3]. In these ES cells, the Venus fluorescence gene was knocked in frame with the first Hes1 exon, resulting in the expression of a Venus–Hes1 fusion protein from the endogenous Hes1 promoter. This method allows the identification and physical separation of Hes1 protein-high and Hes1 protein-low ES cells based on fluorescence intensity using a cell sorter. Following cell sorting, Hes1-high and Hes1-low ES cells were cultured in neural differentiation medium without LIF and BMP. Hes1-high ES cells tended to differentiate into early mesodermal cells associated with brachyury marker expression, even under neural differentiation conditions, and Hes1-low ES cells tended to differentiate into neural cells at an earlier time [3] (Figure 2b). The differences in marker gene expression between Hes1-high and Hes1-low cells were observed a few days after the induction of differentiation, suggesting that Hes1 regulates fate choice depending on the expression level at the initial step of ES cell differentiation and then triggers the phenotypic output over a few days [3]. Because Hes1-null ES cells can keep their pluripotent state in ES cell culture conditions, Hes1 oscillation is dispensable for ES cell culture [3]. However, Hes1 regulates the fate determination step during ES cell differentiation and contributes to the differentiation heterogeneity *in vitro*.

## 6. Differentiation Properties of Hes1-Depleted and Hes1-Sustained ES Cells

We confirmed that the difference in the differentiation properties depends on the level of Hes1 protein expression in ES cells using Hes1-null and Hes1-sustained cell lines cultured in the same neural differentiation medium [3,22]. Hes1-sustained cells contain one additional copy of Hes1 cDNA knocked into the Rosa26 locus and express Hes1 protein at a high level similar to the endogenous maximal level in a sustained manner [22]. Hes1-null ES cells differentiated into neural cells earlier and more uniformly than wild-type ES cells, suggesting that these cells display less heterogeneity of differentiation than wild-type cells [3]. On the other hand, the differentiation of Hes1-sustained ES cells was delayed, and these cells eventually differentiated into early mesodermal cells associated with brachyury expression even under neural differentiating conditions [22,23].

For human pluripotent stem cells, a combination of basic FGF (b-FGF) [24] and ActivinA/TGF-beta signaling [25] is essential to keep the pluripotency, while LIF and BMP signaling are dispensable. Hes1 expression occurs in human ES and induced pluripotent stem (iPS) cells, probably depending on b-FGF, and the expression level is variable in individual cells [26]. We analyzed the neural cell differentiation of human ES and iPS cells [27,28] with or without Hes1 downregulation using shRNA, and found that Hes1 knock-down enhanced neural differentiation to some extent in both human ES and iPS cell lines [26]. However, it remains to be determined whether a similar molecular mechanism is applicable for human pluripotent stem cells.

## 7. The Molecular Mechanism of Heterogeneity in ES Cell Differentiation

It has been previously reported that the inactivation of Notch signaling in ES cells results in preferential cardiac mesoderm differentiation [29–31], whereas forced activation of Notch signaling advances neural differentiation [2]. These reports demonstrated the opposite outcomes of ES cell differentiation of Hes1 level and Notch signal activation. Indeed, Notch signaling, which is required for neural differentiation from ES cells, was completely suppressed in Hes1-sustained ES cells during differentiation [22]. By contrast, the Notch signal was enhanced in Hes1-null cells at an earlier time [3]. These results suggest that the heterogeneous expression of Hes1 is able to set the heterogeneous activation of Notch signaling and that various differentiation patterns depend on the extent to which Notch signaling is active in ES cells [23] (Figure 2b). The major candidate mediating signals from Hes1 to Notch is probably Dll1, a target gene of Hes1 and a ligand of Notch signaling [3]. Dll1 is expressed in signal-sending cells and can transfer the signal to neighboring cells that express the Notch receptor. Hes1 oscillation leads Dll1 oscillation, resulting in various levels of Dll1 expression in individual cells and contributing to various levels of activation of Notch signaling in nearby cells [3]. Hes1-KO cells probably express Dll1 protein uniformly and reciprocally activate Notch signaling in the cell population. Although the precise mechanism by which fluctuating Hes1 expression affects cell fate choice remains to be determined, we speculate that upon the induction of differentiation, Hes1 protein levels could be fixed temporarily, leading to distinct activities of Notch signaling.

Another fluctuating Hes1-target gene, *Gadd45g*, has been reported to be a cell cycle inhibitor during the G2/M phase [32] and a member of the growth arrest DNA-damage-inducible gene 45 (Gadd45) family involved in DNA demethylation and DNA repair [33]. ES cells divide in an unusually short time period [34], and cell cycle exit or cell cycle elongation occurs during their differentiation. We found that Hes1-sustained cells delay initiating their differentiation and maintain the rapid cell cycle longer than wild-type cells [22]. The induction of p57, an indirect target gene of Hes1 and a cell cycle inhibitor at the G1/S phase boundary, is also delayed in Hes1-sustained cells compared to wild-type cells [22]. Moreover, according to the results for Venus-Hes1 ES cells, the Hes1-low population was mostly in the G1 phase of cell cycle [35]. These results suggest that Hes1 oscillation is involved in cell cycle regulation in ES cells. In agreement with this idea, previous reports have revealed a clear correlation between the Hes1 expression level and the cell cycle in many other cell types [10,15,36,37]. For example, it has been reported that Hes1 is mostly absent during early G1 phase and mainly expressed during late G1, S and G2 phase in neural progenitors [10], Hes1 directly or indirectly represses the transcription of cell cycle related genes, p21Cip1, p27Kip1 and p57Kip2 in various types of cells [36–38], and Hes1 controls the reversibility of cellular quiescence of fibroblasts by modifying histone tails and chromatin conformation [39]. Moreover, Hes1 oscillation is important for efficient fibroblast cell proliferation [15]. Detailed analysis is required to understand the relationship between Hes1 expression and the cell cycle of ES cells.

We found many target genes of Hes1 by ChIP-chip analysis, demonstrating that Hes1 binds to the promoter regions or introns of many genes related to cell differentiation [3]. Interestingly, about 40% of Hes1 target genes are reported to display bivalent modifications, histone H3 lysine 4 trimethylation (H3K4me3; active modification) and histone H3 lysine 27 trimethylation (H3K27me3; repressive modification), on the promoter regions in pluripotent stem cells [3,40,41]. This bivalent modification is known as a crucial epigenetic mark of ES cells, and it contributes not only to the pluripotency of the ES cells but also to the control of gene expression during differentiation [42]. About 80% of Hes1 target genes are reported to be modified with H3K4me3 [3,41]. However, the role of Hes1 in these epigenetic modifications is still unknown.

## 8. Conclusions

Hes1 oscillation has been reported in many cell types, such as fibroblasts [8], neural progenitors [10], mesodermal progenitors [43] and ES cells [3], and it could play multiple roles in these cells. Signaling that regulates Hes1 expression, the period of Hes1 oscillation, and the target genes of Hes1 are different in each cell type [3,10]. The stability of Hes1 mRNA is longer in ES cells than in fibroblast cells, which causes the difference of time period (2 h in fibroblast cells and 3–5 h in ES cells) for Hes1 oscillation cycle [3]. However, how cells differentially utilize Hes1 oscillation for multiple roles is unknown. We elucidated the role of Hes1 oscillation in ES cell differentiation, and we propose that the oscillation is a biological tool that produces various levels of Hes1 expression in a cell population that is genetically identical. The Hes1 levels govern the activity of Notch signaling in undifferentiated cells and contribute to various potentials for differentiation via Notch signaling activation [3,22]. Other fluctuating molecules could also work together and produce more diverse responses in cells, causing them to differentiate into various cell types from ES cells.
